# Experimental Platform to Study Spiking Pattern Propagation in Modular Networks In Vitro

**DOI:** 10.3390/brainsci11060717

**Published:** 2021-05-28

**Authors:** Yana Pigareva, Arseniy Gladkov, Vladimir Kolpakov, Irina Mukhina, Anton Bukatin, Victor B. Kazantsev, Alexey Pimashkin

**Affiliations:** 1 Neurotechnology Department, Lobachevsky State University of Nizhny Novgorod, 603950 Nizhny Novgorod, Russia; pigareva@neuro.nnov.ru (Y.P.); gladkov@neuro.nnov.ru (A.G.); kolpakov-v@mail.ru (V.K.); mukhinaiv@mail.ru (I.M.); kazantsev@neuro.nnov.ru (V.B.K.); 2 Cell Technology Department, Central Research Laboratory, Privolzhsky Research Medical University, 603005 Nizhny Novgorod, Russia; 3 The Laboratory of Renewable Energy Sources, Alferov Saint-Petersburg National Research Academic University of the Russian Academy of Sciences, 194021 Saint-Petersburg, Russia; antbuk.fiztek@gmail.com; 4 The Laboratory of Bio and Chemosensor Microsystems, Institute for Analytical Instrumentation of the RAS, 198095 Saint-Petersburg, Russia; 5 Neuroscience and Cognitive Technology Laboratory, Center for Technologies in Robotics and Mechatronics Components, Innopolis University, 1 Universitetskaya Str., 420500 Innopolis, Russia; 6 Center for Neurotechnology and Machine Learning, Immanuel Kant Baltic Federal University, 14 Nevsky Str., 236016 Kaliningrad, Russia

**Keywords:** microfluidics, dissociated culture, modular neural networks, microelectrode array, synaptic plasticity

## Abstract

The structured organization of connectivity in neural networks is associated with highly efficient information propagation and processing in the brain, in contrast with disordered homogeneous network architectures. Using microfluidic methods, we engineered modular networks of cultures using dissociated cells with unidirectional synaptic connections formed by asymmetric microchannels. The complexity of the microchannel geometry defined the strength of the synaptic connectivity and the properties of spiking activity propagation. In this study, we developed an experimental platform to study the effects of synaptic plasticity on a network level with predefined locations of unidirectionally connected cellular assemblies using multisite extracellular electrophysiology.

## 1. Introduction

Theoretical and in vivo studies show that the modular structure of neural networks with a heterogeneous synaptic architecture provides energy-efficient propagation of spiking patterns, which enables optimal information processing [1,2,3]. New advances in bioengineering provide opportunities for the in vitro modeling of modular networks using patterning [4,5] and microfluidic methods [6,7,8,9]. The guidance of axon growth through asymmetric microchannels can be used to form unidirectional connectivity between neuronal networks grown in microfluidic chips [10,11,12,13,14,15,16,17]. The number of axons passing through microchannels regulates the transmission and the connectivity weight of neuronal modules [18,19,20]. Microelectrode arrays combined with microfluidic chips provide electrophysiological monitoring of spiking pattern generation and propagation within and between modular networks [20,21,22,23,24,25]. Such networks demonstrate a large number of spontaneous spiking patterns in contrast to uniform networks [26]. In cortical cultures, modularity enhances the classification of information, as represented by patterns of precise spike timings induced by local electrical stimulation [27]. Recently, a high similarity between close-in-time spontaneous activity patterns was observed in modular networks, suggesting instantaneous memory-like phenomena [24].

Here we developed and studied two types of microfluidic chips with asymmetric microchannels of various designs, which provide unidirectional connections between neuronal networks located in different chambers (modules). The resulting network demonstrates the unidirectional transmission of spontaneous and stimulus-evoked spiking activity between modules. We developed an in vitro platform to monitor and control the network-level effects on the synaptic plasticity induced by the connections between the networks. Unidirectional connectivity between neuronal networks allows the delivery of an electrical stimulus to the pre- or postsynaptic neurons and the evaluation of spiking activity propagation provided by a given connection [28]. In our experiments we show how such modular network technology can provide morpho-functional organization to generate reproducible patterns of spiking activity that can be further used in studies of network-level effects on brain activity.

## 2. Materials and Methods

### 2.1. Microfluidic Device Fabrication

The microfluidic chips were fabricated using polydimethylsiloxane (PDMS) molding techniques. A standard two-layer lithography was used for mold fabrication. For details regarding this process, see [29]. The microfluidic chip consisted of two chambers (220 μm height) for the culturation of neuronal cells and 16 microchannels (5.5 μm height) for neurites growth. According to the predefined direction of axon growth, the subnetworks were labeled Source and Target modules. We studied two types of microchannels: Fish and Octopus. Previously, we showed that the microchannels, which consist of a sequence of triangular shapes, enable unidirectional axon growth between neurons [23]. Increasing the size of the last triangular segment also increased the efficiency of unidirectional axon guidance [30]. The Fish type consisted of three segments: two segments had a narrow triangular shape (200 μm length, 40 μm width), and one triangular segment was larger (200 μm length, 150 μm width) with rounded borders. The large segment had two trap sections to gather the axons of the target module. The width of the bottleneck in the first small triangular segments was 7 μm. The width of the outlet of the microchannels was 30 μm. The Octopus type also consisted of two parts. The first segment consisted of five microtunnels converged into one (15 μm width). This structure was supposed to facilitate axonal growth from the Source module to the Target module. The second segment had a 300 μm length, 150 μm width, and two traps for the axons growing in the opposite direction.

PDMS (Sylgard 184, Dow Corning, Midland, MI, USA) Base and Curing agent were mixed (10:1) and cured in a master mold at 80 °C for 2 h. After peeling, the PDMS was kept in an oven at 100 °C for 12 h to finalize the cross-linking of uncured oligomers [31].

**Figure 1:**
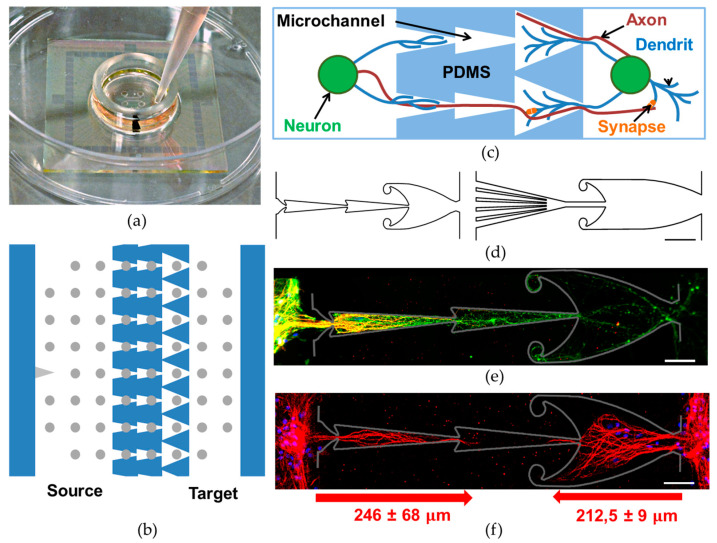
PDMS chip to grow modular neural networks. (**a**) Microelectrode array mounted with microfluidic PDMS chip. (**b**) Schematic view of the microfluidic device mounted to the MEA, with 16 micro-channels connecting the two chambers. Microchannel length was 600 μm in the Fish chip and 700 μm in the Octopus chip. (**c**) Specific microchannel structure provides unidirectional neurite growth. (**d**) Schematic view of microchannels in the Fish chip (on the left) and the Octopus chip (on the right), scale bar 100 μm. (**e**,**f**) Immunofluorescence images from a hippocampal culture at 21 DIV only in the Source chamber: neurons (b3-tubulin, green), neuronal somas and dendrites (Map2, red) and cell nuclei (DAPI, blue) (**e**) and in both the Source and the Target chambers—neuronal somas and dendrites (Map2, red) and cell nuclei (DAPI, blue), scale bar 50 μm (**f**). Chips were removed. Gray lines indicate the manually marked previous locations of the microchannel boundaries. The arrows below indicate the average length of dendrites growing from the Source and the Target modules.

The wells for cell seeding were cut with a puncher (2 mm in diameter) at two opposite corners of each chamber. The chips were cleaned with compressed air (2 kPa, filters 1 and 0.05 μm) and 3M Scotch (3M, St. Paul, MI, USA). We mounted the PDMS chips with microelectrode arrays (MEAs) to study the electrical activity of the cultures. The chips were manually aligned with the MEAs, which were composed of 60 electrodes (TiN electrodes, diameter 30 µm with 200 µm in-between, Multichannel Systems, Reutlingen, Germany) under a binocular, so that each of 8 microchannels encompassed 3 electrodes (Figure 1b). A drop of 96% ethanol was applied before the alignment. The PDMS chips were also bonded to the coverslips for immunostaining analysis of the neurites.

The surfaces of the prepared PDMS devices were coated with the adhesion-promoting molecule polyethyleneimine (PEI, 1 mg/mL, Sigma-Aldrich, P3143, Saint Louis, MI, USA) at 4 °C overnight. The PEI was washed three times with deionized water. Subsequently, the chips were filled with laminin (20 μg/mL, Sigma-Aldrich, L2020, Saint Louis, MI, USA) and incubated at 37 °C for 30 min.

### 2.2. Cell Culturing

Hippocampal cells were dissociated from embryonic mice (E18) and plated in the cell chambers of the PDMS chips at an initial density of approximately 7000–9000 cells/mm^2^. Cells were removed from the seeding wells using a pipette tip the next day and remained only in the cell chambers. Experiments were carried out following the protocol № 23 (31.10.2018) approved by the Bioethics Committee of the National Research Lobachevsky State University of Nizhny Novgorod (Russia). Additional regulations included Order No.199n “On Approval of the Rules of Good Laboratory Practice” (Russia, 2016) and Directive 2010/63/EU of the European Parliament and the Council of the European Union (22.09.2010) on the protection of animals used for scientific purposes. For additional culturing procedure details, see [32]. The cells were seeded in Neurobasal medium (Invitrogen, 21103049, Carlsbad, CA, USA) enriched with 2% Supplement B-27 (Invitrogen, A3582801, Carlsbad, CA, USA), 1% glutamine (Invitrogen, 25030-024, Carlsbad, CA, USA), 5% fetal calf serum (Invitrogen, A3160801, Carlsbad, CA, USA), and gentamicin 20 μg/mL (AppliChem, A1492, Darmstadt, Germany). On the next day, half of the medium was replaced with a new one, in which the cells were cultured. The cells were cultured under constant conditions of 35.5 °C, 5% CO_2_, in a humidified cell culture incubator (3552-2, SHEL LAB, Cornelius, OR, USA). The cells were cultured in Neurobasal Plus medium (Invitrogen, A3653401, Carlsbad, CA, USA) enriched with 2% Supplement B-27 (Invitrogen, A3653401, Carlsbad, CA, USA), 1% glutamine (Invitrogen, 25030-024, Carlsbad, CA, USA), 0.4% fetal calf serum (Invitrogen, A3160801, Carlsbad, CA, USA) and gentamicin 20 μg/mL (AppliChem, A1492, Darmstadt, Germany). Half of the medium was replaced every two days.

### 2.3. Immunostaining

For immunostaining, we used cultures grown in microfluidic Fish chips bonded to the coverslips. At 21 DIV, the neuronal cultures were taken for immunostaining. The chips were mounted using a reversible method and were removed to access the neurites for staining. The culture medium was first replaced with warm (37 °C) PBS (Invitrogen, 10010023, Carlsbad, CA, USA). Then, the cells were fixed with warm (37 °C), freshly prepared 4% paraformaldehyde (Sigma-Aldrich, P6148, Saint Louis, MI, USA) for 15 min at room temperature and then washed with PBS three times for 5 min. The cells were permeabilized with 0.1% Triton X-100 (Sigma-Aldrich, X100, Saint Louis, MI, USA) in PBS with 2% BSA (Bovine Serum Albumin) for 20 min. Primary antibodies consisted of βIII-tubulin (guinea pig, 1:700, SYSY, 302 304, Göttingen, Germany) and Map2 (chicken, 1:1000, Abcam, ab92434, Cambridge, UK). The cells were incubated with primary antibodies in PBS with 1% BSA at 4 °C overnight. Then we rinsed the cultures three times with PBS for 5 min. Goat anti-Guinea Pig Alexa Fluor 647 (1:400, Thermo Fisher Scientific, A-21450, Waltham, MA, USA) and Goat anti-Chicken Alexa Fluor 546 (1:400, Thermo Fisher Scientific, A-11040, Waltham, MA, USA) secondary antibodies were used for β3-tubulin and Map2, respectively. Hoechst 33342 (1:350, Abcam, ab228551, Cambridge, UK) was added along with secondary antibodies to label cell nuclei. The cells were incubated in the dark with secondary antibodies in PBS with 5% BSA at room temperature for 60 min and then rinsed with PBS and deionized water to remove the salt. After that, the cells were fixed in a mounting medium (Sigma-Aldrich, Saint Louis, MI, USA).

### 2.4. Neurite Analysis

Light field images were acquired with an Axio Observer A.1 microscope fitted with an Axiocam ICm1 camera (Zeiss, Jena, Germany) on DIV 3, 4, 5, and 10. Fluorescence was observed using a confocal laser scanning microscope from Zeiss (LSM 510). Images were analyzed with ImageJ software. The lengths of the dendrites extending into the microchannel were measured at a maximal distance from the edge of the culturing module to the end of the marked dendrites within the microchannel.

### 2.5. Electrophysiology

Spiking activity was recorded from 59 (1 reference) TiN electrodes of the MEA system (Multichannel Systems, Reutlingen, Germany) at a sample rate of 20 kHz. Detection of the recorded spikes was based on the threshold calculation of the signal median. Details of the spike and burst detection methods were described in our previous study [32]. All signal analysis and statistics were performed with custom-made software in Matlab (R).

Spontaneous activity was recorded every 5 days from 10 DIV until 25 DIV. Probabilities of burst propagation in a forward direction and the opposite direction were calculated as previously described [23]. The direction of the burst propagation was based on a time delay between the maximum instantaneous (1 ms bin) spiking rate of the bursts. The probabilities of burst propagation in a forward direction and the opposite direction were estimated as the number of bursts propagated from the Source to the Target divided by the burst number in the Source, and the bursts propagated from the Target to the Source divided by the burst number in Target, respectively.

The relative difference between mean burst duration and burst propagation was calculated by dividing the corresponding parameter value for the Octopus and the Fish chips (O/F).

### 2.6. Stimulation Protocol

Stimulation of the electrodes on the MEA was performed using the STG-4004 stimulator (Multichannel Systems, Reutlingen, Germany). A low-frequency test stimulation consisted of 60 or 300 stimuli with a 1–5 s interstimulus interval which varied according to the spontaneous burst frequency. Each pulse was bi-phasic, ±800 mV, and the duration of each phase was 260 µs, positive first. The test stimulation was applied to four selected stimulation sites (electrodes) in four adjacent microchannels to evaluate the microchannel efficiency of postsynaptic activation in the neurons of the Target module.

### 2.7. Plasticity Induction Stimulation

Stimulation protocol consisted of the following steps:

1. Test stimulation of a presynaptic part of a modular network simultaneously applied to four selected stimulation sites (electrodes) in four adjacent microchannels with 1–5 s interstimulus intervals depending on the spontaneous burst frequency (test 1);
2. Repetition of the test stimulation to estimate the spontaneous changes of the responses (test 2);
3. Tetanic stimulation consisting of 20 trains of 10 stimuli with a 50 ms interval between pulses and a 5 s interval between the trains [33] was delivered simultaneously to the same four electrodes in the adjacent microchannels;
4. Repetition of the test stimulation to estimate plasticity-induced changes in spiking activity (test 3).

All plasticity induction experiments were performed on 26–28 DIV.

### 2.8. Analysis of Stimulus Responses

Stimulation-evoked responses in the form of a burst of spikes were recorded from the group of electrodes. To characterize the evoked bursts, we used a post-stimulus time histogram (PSTH) [34]. We estimated the histogram of spiking times in response to each stimulus for all the recording electrodes in the module, and normalized it to the number of stimuli. For Fish cultures the PSTH was estimated at 10–200 ms intervals after stimulation with a 2 ms time-bin. For Octopus cultures, the PSTH was estimated at 10–110 ms intervals after stimulation with a 2 ms time-bin.

The PSTH difference (PRE) and (POST) was calculated separately for each recording electrode in the Target module as follows: mean PSTH (test 2)–mean PSTH (test 1) and mean PSTH (test 3)–mean PSTH (test 2), respectively. Mean PSTH was calculated by averaging the PSTH differences of all the electrodes from the Source module and the Target module separately. The averaged magnitude of the PSTH was estimated by averaging the absolute values of the PSTH differences after tetanic stimulation (test 3 and test 2) over selected electrodes (Source or Target module) for the experiments in which a significant mean PSTH difference between PRE and POST was found (Mann–Whitney Rank Sum Test, *p* < 0.05).

## 3. Results

First, we investigated neurites’ growth in two types of microchannels. The Fish shape had a microchannels structure as shown in Figure 1d, left (see Methods for more details). The second type (the Octopus shape) had microchannels with a higher level of asymmetry (Figure 1d, right). Five microtunnels with a total cross-section area of (7µm × 5 × 5.5 µm) 192.5 μm^2^ collected the neurites from the Source module and converged to one with a cross-section area of (15 µm × 5.5 µm) 82.5 μm^2^. Axons from the Source module grew through this channel to the large section, which contained the axons and the dendrites from the Target module. The shape of the large section was similar to the Fish chip, but was 100 μm longer. Neurites from the Source module grew into the large section (400 µm) in the Octopus channels for 2.5 ± 0.5 days, and in the Fish channels for 4 ± 0.5 days (mean ± std, *n* = 24 and 22 channels, respectively, 2 chips of each type, (Mann–Whitney Rank Sum Test, *p* < 0.05)). The rapid filling of the microchannel bottleneck with axons from the Source module could be important for unidirectional connection between modules.

Then, we studied the maximum axon and dendrite growth length in the microchannels if the cells were seeded only in the Source chamber. The cultures were stained at 21 DIV with an antibody against the structural protein neuronal microtubules b3-tubulin (Figure 1e, green) to mark the whole cell, Map2 to mark the dendrites and somas (Figure 1f, red), and cell-permeable DNA-binding dye DAPI to highlight the soma compartment (Figure 1e, blue). The dendrites (Figure 1e, yellow) grew into the first and second sections of the microchannels (280 ± 70 μm, *n* = 31 channels) while the axons grew throughout the whole microchannel (Figure 1e, green). Staining of the cells in both the modules showed that the dendrites from the Source (246 ± 68 μm, *n* = 73 channels) did not overlap with the dendrites originating from the Target (212.5 ± 9.0 μm, *n* = 3 channels) on 21 DIV (Figure 1e).

To study the spiking activity, the microfluidic chips were mounted to the MEAs (see Methods). The bursting activity of the Fish microchannels (11 MEAs) and the Octopus microchannels (5 MEAs) was recorded from 10 DIV to 25 DIV every 5 days. Spontaneous bursts were observed in both modules of 10 cultures with Fish structures and 4 cultures with the Octopus. In other cultures, bursting activity did not appear in one or both modules, so these cultures were excluded from further analysis.

**Figure 2:**
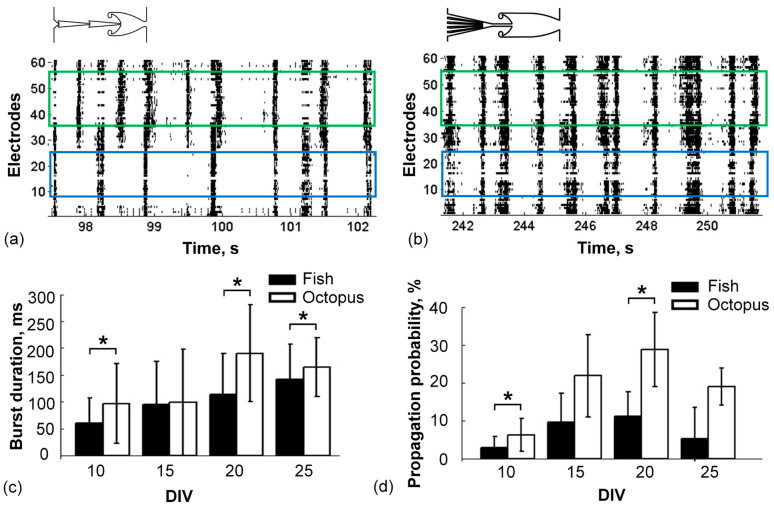
Propagation of spontaneous bursts between modules in two types of microchannels. (**a**) Raster plot of the activity in the Fish chip (see Methods for details). Some of the bursts propagated from the Source to the Target module. A blue box indicates the Source module, green box—the Target module. (**b**) Raster plot of the activity in the Octopus chip. Most of the bursts propagated from the Source to the Target module. (**c**) Mean (± standard deviation) burst durations recorded in the Source module of the Fish (black, *n* = 10 cultures) and the Octopus chip (white, *n* = 4 cultures) during culture development from 10 DIV to 25 DIV. (**d**) Probability of burst propagation from the Source to the Target module in the Fish and the Octopus chips. Mann–Whitney test, *p* < 0.05 (*).

We compared the durations of spontaneous bursting activity of two specific microchannel types. The Target module had various synaptic inputs from the Source module, depending on the microchannel shapes and length, which could influence, e.g., modulate, the activity during the development, so only the bursts in the Source module were analyzed. The burst duration in the Fish cultures monotonically increased from 60 ± 46 ms on 10 DIV to 141 ± 65 ms on 25 DIV (mean ± std, *n* = 10 cultures, Kruskal–Wallis One Way Analysis of Variance on Ranks, *p* < 0.001; Figure 2c). Similarly, in the Octopus cultures, the activity increased from 96 ± 74 on 10 DIV to its maximum of 190 ± 90 on 20 DIV (mean ± std, *n* = 4 cultures, Kruskal–Wallis One Way Analysis of Variance on Ranks, *p* < 0.001; Figure 2c). The maximum relative difference of the mean durations of O/F was 1.56 and was observed on 20 DIV. Then, we estimated the percentage of spontaneous bursts propagated from the Source to the Target modules (see Methods) during development. The propagation was within the range of 3 ± 3–11 ± 6.5% for the Fish and 6.3 ± 4–28 ± 9.7% for the Octopus cultures, which were 2.5 times more efficient on average during the development, and the maximum relative difference was equal to 3.5 on 25 DIV. The relative difference of the mean propagations of O/F was 2.60 on 20 DIV. Statistically significant differences between the propagations were observed on 10 and 20 DIV (Mann–Whitney Rank Sum Test, *p* < 0.05).

The propagation of the bursts in the opposite direction did not exceed 1.6 ± 6% for the Fish chips and was not observed in the Octopus chips.

**Figure 3:**
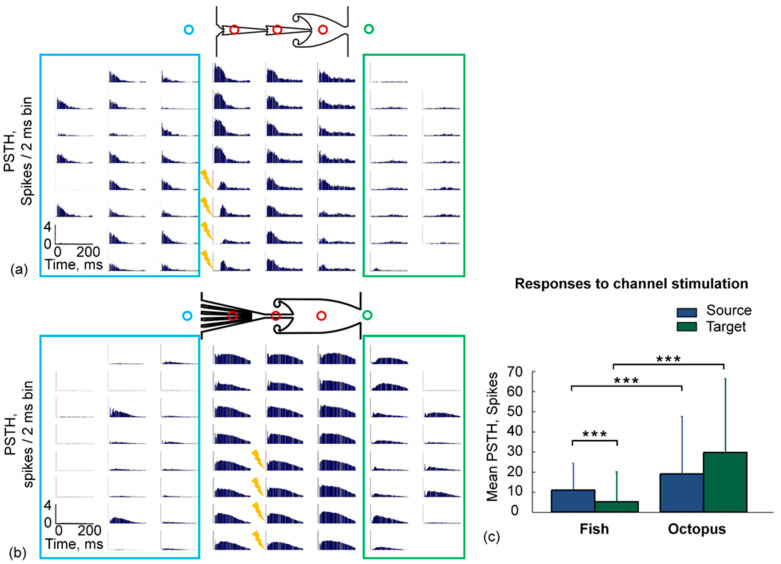
Spiking patterns in response to low-frequency test stimulation applied to the axons in the microchannels. PSTH map of the responses induced by test stimulation (see Methods) of four electrodes in microchannels in Fish (**a**) and Octopus (**b**) chip. (**c**) The mean PSTH of responses in the Source and the Target modules to electrical stimulation of four microchannels. Mann–Whitney Rank Sum Test, *p* < 0.001 (***).

Next, we applied electrical stimulation to the axons in the microchannels to evoke postsynaptic responses in the Target module. We used a train of test stimuli applied to four microchannels simultaneously (see Methods). We found that each stimulus depolarized the axons and evoked a bursting response in both the Target and the Source modules. Bursting activity in the Source module might be caused by retrograde spike propagation in both kinds of the chips (Figure 3a,b). Figure 3 depicts the post-stimulus time histogram (PSTH) of the responses in each electrode during the test stimulation in the Fish culture (a) and the Octopus culture (b). The average number of stimulus-evoked spikes (mean PSTH) on the electrodes of the Source module was equal to 11 ± 13 (*n* = 5 cultures, 10 experiments) and 19 ± 28 (*n* = 4 cultures, 6 experiments) in the Fish and Octopus cultures, respectively (Mann–Whitney Rank Sum Test, *p* < 0.001; Figure 3c, blue bar). We found that the mean PSTH in the Target module of the Fish cultures was equal to 5.3 ± 15, which was lower than that obtained in the Source module (Mann–Whitney Rank Sum Test, *p* < 0.001). On the other hand, evoked activity in the Octopus chip in the Target module was similar to the Source module (Mann–Whitney Rank Sum Test, *p* = 0.5). On average, responses in the Octopus chips were significantly higher compared to the Fish chips (Mann–Whitney Rank Sum Test, *p* < 0.001; Figure 3c, green bars).

Finally, we applied tetanus stimulation (see Methods) to the axons grown in the microchannels and analyzed the change in test stimulus responses in the Source (presynaptic) and the Target (postsynaptic) modules.

**Figure 4:**
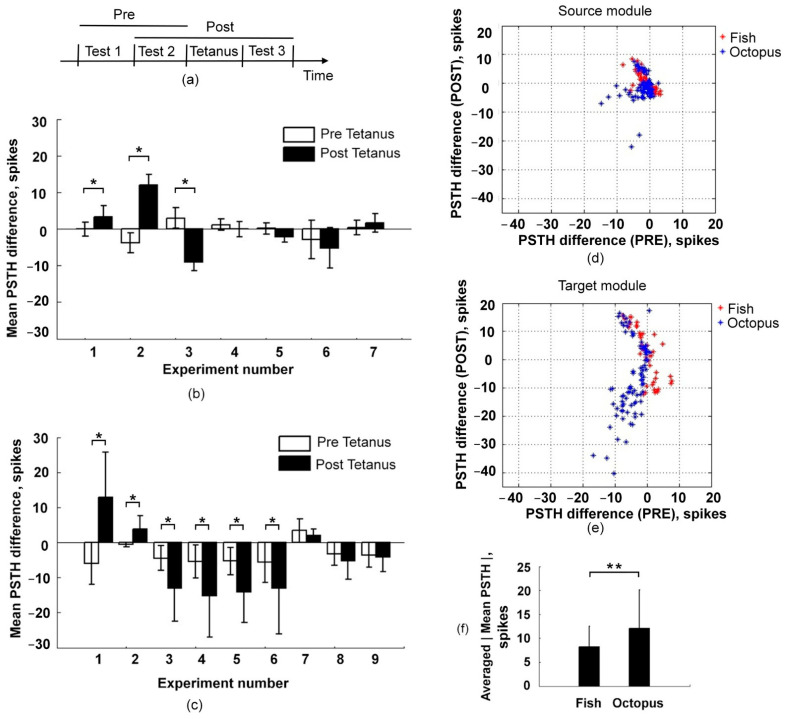
Tetanus-induced changes in evoked responses. (**a**) Schematic illustration of the experimental protocol. (**b**,**c**) Mean of PSTHs difference of the responses in pre- (white bar) compared with the mean difference in post-tetanus (black bar) (see Methods) in Fish (*n* = 7 experiments, 3 cultures) (**b**) and Octopus chips (*n* = 9 experiments, 4 cultures) (**c**). Mann–Whitney Rank Sum Test, *p* < 0.05 (*). (**d**,**e**) Scatter plot of the PSTH difference in each electrode for pre- and post-tetanus of the Fish (red) and the Octopus (blue) chips for the Source (**d**) and the Target module (**e**). Only experiments with a significant effect of tetanization are shown. (**f**) The averaged absolute values of mean PSTH after tetanic stimulation (see Methods) of cultures in Fish (*n* = 3 experiments) and Octopus chips (*n* = 6 experiments). Mann–Whitney Rank Sum Test, *p* < 0.01 (**).

We found that in the Fish chips, the PSTH difference (see Methods) in the Target module after tetanus was significantly greater than before in two samples, indicating potentiation, and in one instance was significantly lower than before tetanus, indicating synaptic depression (Figure 4b, *n* = 7 experiments, 3 cultures, Mann–Whitney Rank Sum Test, *p* < 0.05). In the Octopus chips the depression effect was found in four samples and the potentiation effect was detected in two samples from nine (Figure 4c, *n* = 9 experiments, 4 cultures, Mann–Whitney Rank Sum Test, *p* < 0.05). In summary, a significant effect of the tetanization was found in the Fish chips in three out of seven experiments (42%) and in the Octopus chips in six out of the nine experiments (67%).

Analysis of the PSTH difference for individual electrodes showed various magnitudes of the effect for the Octopus (Figure 4e, blue) and the Fish chips (Figure 4e, red) (Mann–Whitney Rank Sum Test, *p* < 0.01). The averaged magnitude of the PSTHs (see Methods) as absolute values of PSTH differences averaged for the experiments where tetanization effects were observed in the Octopus chips was 12 ± 8 spikes (*n* = 4 cultures, 6 experiments) and for the Fish chips was 8 ± 4.3 spikes (*n* = 2 cultures, 3 experiments).

In the Source module the averaged magnitude of the PSTHs after tetanus (Figure 4d) was significantly less than in the Target chamber (Mann–Whitney Rank Sum Test, *p* < 0.01), and the averaged magnitude of PSTH values for the Octopus chips was 2.8 ± 3.2 spikes and for the Fish chips was 2.5 ± 1.9 spikes.

## 4. Discussion

We have shown how modular neuronal circuits in vitro, generating reproducible spiking patterns with desired characteristics, can be designed and fabricated using microfluidic microchannel technology. We proposed two types of microfluidic chips that provided different strengths of connectivity between two neuronal cultures. Specific microchannel geometry enabled the formation of unidirectional connectivity and spiking activity propagation predominantly moving from the Source to the Target. Analysis of network spiking activity using microelectrode arrays revealed that two types of microchannels provided relatively weak and strong connectivity, which can be used to model and study various modular structures of the brain. We showed that the Octopus type of chips provided significantly higher stimulus responses in Target modules than the Fish chips. In addition, such chips provided a higher propagation of spontaneous spiking activity between modules.

Precisely aligned microelectrodes placed on the microchannels allow the depolarization of axons growing from the source module, with electrical stimuli that synaptically evoked the activity in the target module. High-frequency electrical stimulation of such electrodes allows the induction and study of synaptic plasticity throughout the whole network. A similar approach using tetanus stimulation, previously proposed by Jimbo [33], was applied to single electrodes in the homogeneous networks of cultured neurons. Such an approach induced “pathway-specific plasticity” and potentiated or depressed several neuronal connections. The results were replicated by several other laboratories with tetanus or other modified stimulation protocols [34,35,36]. Our approach, using modular networks, focuses on plasticity changes in predefined connectivity pathways grown through the microchannels. 

## 5. Conclusions

In summary, we developed two types of microfluidic chips that formed modular network structure of dissociated cultured cells with various strengths of connectivity and unidirectional spiking patterns propagation. Such experimental model also can address new questions of plasticity timescale effects up to several days or weeks, and the network-wide spatial scale effects of heterosynaptic plasticity in various types of neurons.

We believe that our modular neuronal circuits in vitro with controllable structural and functional organization can also be useful in preclinical studies of the network-level effects of pharmacological and other stimulations on network-level neuronal signaling. Another prospective application could be the design of living-cell information processing systems trained to generate certain input–output functions similarly to artificial neuronal networks (ANNs).

## Data Availability

The data presented in this study are available on request from the corresponding author.
